# Arteriogenesis and Therapeutic Angiogenesis—An Update

**DOI:** 10.3390/ijms222413244

**Published:** 2021-12-09

**Authors:** Elisabeth Deindl, Paul H. A. Quax

**Affiliations:** 1 Walter-Brendel-Centre of Experimental Medicine, University Hospital, Ludwig-Maximilians-Universität, 81377 Munich, Germany; 2 Biomedical Center, Institute of Cardiovascular Physiology and Pathophysiology, Ludwig-Maximilians-Universität München, Planegg-Martinsried, 82152 Munich, Germany; 3 Department of Surgery, Einthoven Laboratory for Experimental Vascular Medicine, Leiden University Medical Center, 2300 RC Leiden, The Netherlands

Vascular occlusive diseases such myocardial infarction, peripheral artery disease of the lower extremities, or stroke still represent a substantial health burden worldwide. In recent times, they have come even more into focus as thromboembolic events associated with vascular occlusive diseases are known to belong to the severe complications observed in patients with SARS-CoV-2 infection [1]. To understand the mechanisms of blood flow recovery in terms of arteriogenesis and therapeutic angiogenesis is a major goal in order to develop efficacious non-invasive treatment options for afflicted.

This Special Issue of the *International Journal of Molecular Sciences* entitled “Arteriogenesis and Therapeutic Angiogenesis” follows up with recent advances that are specific to that field of research.

One of the most important points is to identify vascular occlusive diseases already in their beginning or early progression, enabling clinicians to induce natural bypass growth—a process that is defined as arteriogenesis or that is referred to as therapeutic angiogenesis—in time [2,3]. Since patients who are at that stage are often asymptomatic, this represents a considerable challenge. In the current issue, Saenz-Pipaon et al. describe the need for reliable biomarkers for peripheral artery disease (PAD), whereby they focus on the lower limbs. PADs have a high prevalence, show a poor prognosis, and are associated with a high risk of myocardial infarction and stroke. In their article, Saenz-Pipaon et al. discuss the appropriateness of inflammatory molecules, liquid biopsies, and non-coding RNAs and even focus on the potential of machine learning methods [4].

Mohamed Sabra and colleagues describe the mechanisms of vascularization, i.e. vasculogenesis, angiogenesis, and arteriogenesis, and critically elucidate the usefulness of angiogenic therapies such as protein therapy, gene therapy, stem cell therapy, and extracellular vesical therapy. Moreover, they address the relevance of patient selection and delivery methods, and introduce bioinformatics and bioengineering as promising future tools for the treatment of patients with vascular occlusive diseases [5]. The paper by Beltrain-Camacho focusses on angiogenic cell therapy as a treatment option for critical limb ischemia, and also the use of microRNAs, exosomes, and secretomes are briefly discussed [6].

The article by Ashraf and Zen is more oriented towards basic science and deals with the function of the quiescent contractile and the proliferative synthetic phenotypes of a vascular smooth muscle cell (VSMC) as well as the molecular mechanisms and regulations of phenotype switching. Moreover, the article critically addresses the option to target phenotype switching in patients aiming to promote arteriogenesis [7].

Zeen Aref and Paul Quax highlight the difficulty of investigating the mechanisms of angiogenesis in hind limb ischemia models that are associated with arteriogenesis. They introduce an in vivo Matrigel plug assay, which is superior to current hind limb models, as it allows the analysis of ischemia-induced angiogenesis without the influence of collateral artery growth that is occurring in parallel [8]. Besides arteriogenesis also the mouse strain chosen has a major influence on the outcome of angiogenesis. Kübler et al. address this topic and explain the relevance of selecting the appropriate mouse stain depending on the scientific question that is asked. In particular, they focus on the influence of C57BL/6J and SV-129 strain-related differences in leukocyte recruitment in ischemic angiogenesis [9].

Cold-inducible RNA-binding protein (CIRP or CIRBP) is a stress inducible protein that contains RNA and protein binding domains and that has recently come into the focus of vascular research [10,11,12]. In the current issue of the *International Journal of Molecular Sciences,* two groups have independently shown that the absence of CIRP promotes angiogenesis in ischemic muscle tissue, and, interestingly enough, they have identified two independent mechanisms. Kübler et al. have demonstrated that the absence of extracellular CIRP results in a reduced number of M1-like polarized pro-inflammatory and an increased number of M2-like polarized regenerative macrophages associated with reduced tissue damage and an increased capillary to muscle fiber ratio [13]. Goossens et al. have identified CIRBP as a negative modulator of angiogenesis through its function to regulate the angiomiRs miR-329-3p and miR-495-3p [14], which have previously been shown to be involved in vascular regeneration [10].

Yoshitomi and colleagues present the role of the AP-1 transcription factor family Jun B in angiogenesis and highlight its function in tip cell formation and tissue-specific vascular maturation [15].

Endoglin is a co-receptor of transforming growth factor-β1 (TGF-β1), and mutations of this transmembrane protein are known to cause the vascular disorder hereditary hemorrhagic telangiectasia type 1(HHT1). By investigating *Eng*+/− mice in a myocardial infarction model, Bakker et al. were able to show that these mice display more M1-like polarized macrophages, whereas the number of M2-like polarized macrophages was reduced. These data somehow reflect data from the clinic, as patients with HHT1 also show an increased number of inflammatory macrophages. Astonishingly, the treatment of *Eng*+/− mice with a bone morphogenetic protein (BMP) receptor kinase inhibitor improved heart function and vascularization, suggesting that the BMP receptor kinase may present a promising therapeutic target for HHT1 patients in the future [16].

Gatina and co-workers investigated the serviceability of recombinant multicistronic mutagenic contructs in terms of safety and efficacy to treat ischemic disease. By using 2A-peptide-based constructs encoding vascular endothelial growth factor (VEGF) and fibroblast growth factor 2 (FGF2), they were able to demonstrate increased levels of the named recombinant proteins along with an increased number of capillary-like structures in genetically modified human umbilical vein endothelial cells (HUVECs) in vitro [17]. Last not least, Baganha et al. investigated the suitability of two-photon intravital microscopy (2P-IVM) to assess the permeability of microvessels in atherosclerotic vein grafts in mice. From their study, they concluded that 2P-IVM is a promising tool that can be used to analyze plaque angiogenesis and leakiness in preclinical atherosclerosis models in vivo [18].

In summary, we think that our new Special Issue on arteriogenesis and therapeutic angiogenesis is a rewarding collection of original and review articles in the field of vascular research that will serve as inspiration for future pioneering investigations looking into the treatment of vascular occlusive diseases.
